# Intracranial Spotty Calcium Predicts Recurrent Stroke in Patients with Symptomatic Intracranial Atherosclerotic Stenosis: A Prospective Cohort Study

**DOI:** 10.1007/s00062-023-01299-7

**Published:** 2023-06-07

**Authors:** Rui Li, Moqi Liu, Jialu Li, Xueqiao Jiao, Xiuhai Guo

**Affiliations:** 1 Department of Neurology, Xuanwu Hospital, Capital Medical University, 100053 Beijing, China; 2 National Center for Neurological Disorders, Xuanwu Hospital, Capital Medical University, 100053 Beijing, China

**Keywords:** Intracranial artery, Ischemic stroke, Spotty calcium, Computed tomography angiography, Stenosis

## Abstract

**Purpose:**

Accumulating evidence highlights the association of calcium characteristics and cardiovascular events, but its role in cerebrovascular stenosis has not been well studied. We aimed to investigate the contribution of calcium patterns and density to recurrent ischemic stroke in patients with symptomatic intracranial atherosclerotic stenosis (ICAS).

**Methods:**

In this prospective study, 155 patients with symptomatic ICAS in the anterior circulation were included, and all subjects underwent computed tomography angiography. The median follow-up for all patients was 22 months and recurrent ischemic stroke were recorded. Cox regression analysis was performed to examine whether calcium patterns and density were associated with recurrent ischemic stroke.

**Results:**

During the follow-up, 29 patients who experienced recurrent ischemic stroke were older than those without recurrent ischemic stroke (62.93 ± 8.10 years vs. 57.00 ± 12.07 years, *p* = 0.027). A significantly higher prevalence of intracranial spotty calcium (86.2% vs. 40.5%, *p* < 0.001) and very low-density intracranial calcium (72.4% vs. 37.3%, *p* = 0.001) were observed in patients with recurrent ischemic stroke. Multivariable Cox regression analysis showed that intracranial spotty calcium, rather than very low-density intracranial calcium, remained an independent predictor of recurrent ischemic stroke (adjusted hazard ratio 5.35, 95% confidence interval 1.32–21.69, *p* = 0.019).

**Conclusion:**

In patients with symptomatic ICAS, intracranial spotty calcium is an independent predictor of recurrent ischemic stroke, which will further facilitate risk stratification and suggest that more aggressive treatment should be considered for these patients.

**Supplementary Information:**

The online version of this article (10.1007/s00062-023-01299-7) contains supplementary material, which is available to authorized users.

## Introduction

Intracranial atherosclerotic stenosis (ICAS) is one of the common causes of ischemic stroke. In western countries, ICAS accounts for 10–16% of ischemic stroke cases, while in Asia it accounts for up to half of all ischemic stroke cases [1–3]. Given the high rate of recurrent stroke even with aggressive medical management, the role of endovascular procedures in symptomatic ICAS has been the focus of recent multicenter clinical trials, but they were unable to conclude that stenting is superior to medical treatment [4–6]. Up to now, secondary prevention strategies for symptomatic ICAS are challenging. It is desirable to further investigate predictors of stroke recurrence in these patients, for better risk stratification and then to formulate tailored treatment regimens.

Vascular calcification is an important proxy of atherosclerotic disease, while its role is complicated and incompletely understood [7]. Several studies have shown that the presence of calcification in intracranial arteries could be a potential predictor of future ischemic stroke and associated with the prognosis of stroke [8–10]. Contrarily, calcific plaque is generally considered a stable form of atherosclerosis, which is unlikely to rupture and result in ischemic events [11]. Emerging evidence has pointed toward the importance of the association between calcium features and different risks of cardiovascular events [12, 13]. Calcium density exhibited an inverse association with acute coronary syndrome [14–16]. In addition to the calcium density, two distinct calcium patterns, spotty calcium (SC) and large calcific plaque, are likely to have differential effects on clinical risk and outcomes. Numerous studies of coronary arteries have confirmed that SC is related to coronary ischemic events and considered as a predictor of plaque rupture [17, 18]. Recently, two cross-sectional studies reported the potential relationship between SC and ischemic stroke [19, 20]. However, there is a scarcity of clinical data on calcium features in patients with ICAS, as well as the risk of recurrent stroke.

We hypothesized that calcium patterns and density might help to identify symptomatic ICAS subjects with a high risk of recurrent ischemic stroke. This study aimed to provide a method that is easy for physicians to quickly implement for better risk stratification and decision making.

## Methods

### Study Design and Participants

This single-center prospective cohort study conformed to the ethical guidelines of the 1964 Declaration of Helsinki and its later amendments. All protocols were approved by the Institutional Medical Ethics Committee and informed consent was obtained from all participants. Symptomatic ICAS patients who were admitted to the Department of Neurology between January 2017 to June 2021 were consecutively recruited. The inclusion criteria were: (1) age 18–80 years old; (2) symptomatic ICAS referred to acute ischemic stroke in the anterior circulation identified by diffusion-weighted imaging; and (3) ≥ 50% stenosis on the relevant middle cerebral artery or intracranial internal carotid artery, as confirmed by magnetic resonance angiography, computed tomography angiography (CTA), or digital subtraction angiography. The exclusion criteria included: (1) patients with lacunar infarction; (2) coexistent ≥ 50% stenosis of the ipsilateral extracranial carotid artery; (3) non-atherosclerotic intracranial stenosis, such as dissection, vasculitis, moyamoya disease and reversible cerebral vasoconstriction syndrome; (4) evidence of cardioembolism (e.g., atrial fibrillation, mechanical prosthetic valve disease, sick sinus syndrome, dilated cardiomyopathy); (5) contraindications to CTA; and (6) patients received endovascular intervention during follow-up. All patients underwent CTA within 2 weeks of symptom onset to further evaluated the degree of stenosis and calcium characteristics. Patients with < 50% stenosis in the relevant intracranial artery on CTA imaging were also excluded from our study. Clinicians provided treatment for symptomatic ICAS patients according to the guidelines [21]. Patients with 50–69% stenosis and minor stroke were treated with 100 mg aspirin plus 75 mg clopidogrel for 21 days (followed by daily aspirin or clopidogrel alone) and control of stroke risk factors according to the CHANCE trial [22]. Patients with ≥70% stenosis were treated with dual antiplatelet therapy for 90 days according to the SAMMPRIS study [5].

### CTA Imaging Protocol

All CTA examinations were performed using a dual-source 192-slice CT scanner (Somatom Force, Siemens Healthcare, Forchheim, Germany). Patients underwent non-enhanced CT imaging, followed by contrast-enhanced image scanning. The scanning parameters were set as follows: detector collimation 2 × 192 × 0.6 mm; gantry rotation time 0.25 s, pitch 3.2; tube voltage 70–90 kV; tube current by automated tube current modulation (CARE Dose4D, Siemens) using a reference tube current time of 330–450 mAs. Contrast media (Ultravist solution 370 mg I/mL; Bayer Healthcare, Berlin, Germany) was intravenously injected through an antecubital vein via a 20–22-gauge needle using a power injector. A total of 40–50 mL of contrast material was injected at a flow rate of 5 mL/s, followed by 60 mL of saline solution. Images were reconstructed using a slice thickness of 0.75 mm and an interval of 0.40 mm. Volume-rendered, maximum intensity projection, multiplanar reformatted, and curved planar reformatted images were generated to assess the carotid, cerebrovascular, and coronary arteries.

### Image Analysis

Calcium characteristics were evaluated by consensus review of two radiologists blinded to the clinical details. Atherosclerotic calcification was defined as a hyperdense region along the artery with CT attenuation ≥ 130 Hounsfield units (HU). The presence of calcification was measured in the coronary artery, aortic arch, extracranial carotid artery, and intracranial artery. Coronary artery included the left main, left anterior descending, left circumflex, and right coronary arteries. The aortic arch was assessed from the fused curved surface of the ascending and descending aorta to the origin of the left subclavian artery, the left common carotid artery, and the brachiocephalic trunk. The carotid artery was divided into C1–C7 segments according to the Bouthillier method [23]. The extracranial carotid artery was measured from the beginning of the common carotid artery to the C1 segment of the internal carotid artery. The intracranial artery was evaluated as the C2‑7 segment of the internal carotid artery, anterior cerebral artery, and middle cerebral artery. The calcium patterns were divided into SC and large calcific plaque according to the size of the calcific plaque. SC was defined as a calcific plaque with a maximum diameter of < 3 mm in any direction, and large calcific plaque was defined as a calcific plaque ≥ 3 mm in size [24]. Calcium density was measured on non-enhanced axial images with a slice thickness of 0.75 mm and a region of interest (ROI) of 0.5 mm^2^. The highest CT value for each calcified plaque was recorded and defined as the calcium density by manually placing the ROI in all planes across the calcified plaque (Supplementary Fig. 1). The intracranial calcium was categorized into four groups according to quartiles of calcium density at baseline CTA imaging, with quartile 1 (Q1) for very low-density calcium (< 632 HU), quartile 2 (Q2) for low-density calcium (632–971 HU), quartile 3 (Q3) for high-density calcium (971–1316.5 HU), and quartile 4 (Q4) for very high-density calcium (≥ 1316.5 HU).

### Follow-up

After CTA imaging at baseline, all patients were followed-up for at least 1 year. The clinical outcome was defined as recurrent ischemic stroke. All patients were followed up by telephone or routine medical outpatient clinic attendance and inquiring whether patients had experienced recurrent ischemic stroke in the past. If patients did not respond to the follow-up, we will find their medical records and try to contact their relatives for more information about the patient’s condition.

### Statistical Analysis

Continuous variables were presented as mean ± standard deviation or median (interquartile range, IQR). The Mann-Whitney U test and the t‑test were used to compare continuous variables between groups. Categorical variables were expressed as numbers (proportions) and compared using the χ^2^-test or Fisher’s exact test. Variables were included for multivariate analysis if they were *p* < 0.1 in the univariate analysis. Multivariable Cox proportional hazards regression was used to examine the association of calcium patterns and density with recurrent ischemic stroke. Cumulative event-free curves were constructed for recurrent ischemic stroke by the Kaplan-Meier method. All tests were 2‑sided, and *p* < 0.05 was considered statistically significant. All statistical analyses were performed with SPSS statistical software, version 25.0 (IBM, Armonk, NY, USA).

## Results

### Patient Characteristics

**Fig. 1:**
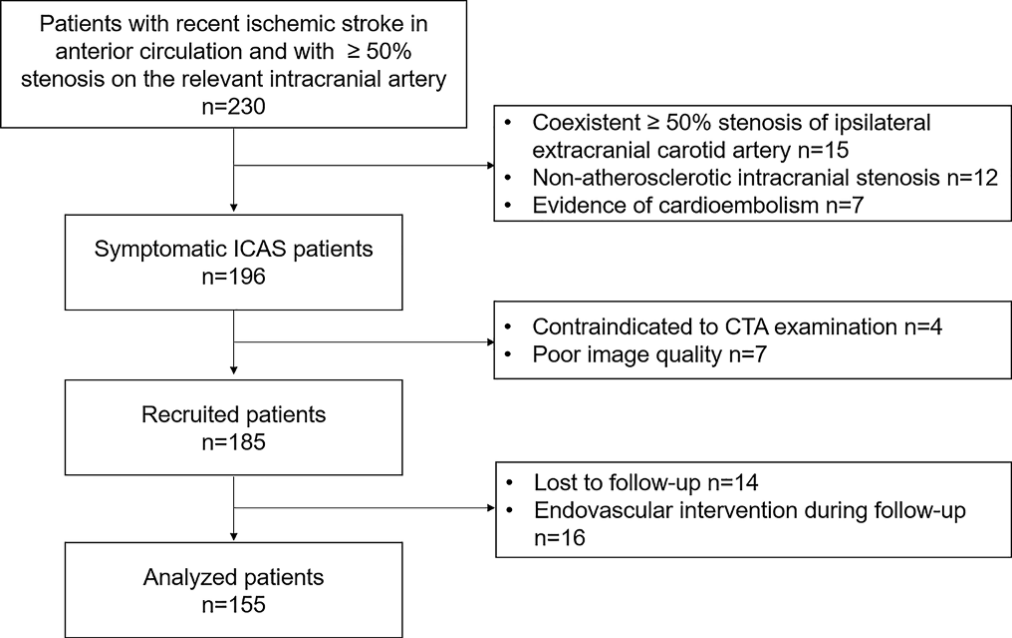
Flowchart of patients enrolled. *CTA* computed tomographic angiography

Of 230 patients with recent ischemic stroke in the anterior circulation and with ≥ 50% stenosis on the relevant intracranial artery, 196 were diagnosed with symptomatic ICAS. A total of 41 patients were excluded, including 16 patients who received preventive endovascular intervention during follow-up, 14 patients who were lost to follow-up, 7 patients with poor image quality, and 4 patients with contraindications to CTA examination. The flowchart of enrolled patients is shown in Fig. 1. A total of 155 patients (age 58.11 ± 11.64 years; 112 males) were included in the final analysis. The median follow-up was 22 months (IQR 19.6–24.3 months), During the follow-up, 29 patients (18.7%) experienced recurrent ischemic stroke. The clinical data are detailed in Table 1. Patients with recurrent ischemic stroke were older than those without recurrent ischemic stroke (62.93 ± 8.10 years vs. 57.00 ± 12.07 years, *p* = 0.027). No significant differences were found in other cardiovascular risk factors and lipid profiles between these two groups.

**Table 1:** Demographic and clinical characteristics of patients with and without recurrent stroke

	Recurrence (*n* = 29)	No recurrence (*n* = 126)	*p* value
Age (years), mean ± SD	62.93 ± 8.10	57.00 ± 12.07	0.027
Male, *n* (%)	21 (72.4%)	91 (72.2%)	0.983
BMI (kg/m^2^), mean ± SD	25.33 ± 3.08	25.39 ± 3.19	0.929
Smoking, *n* (%)	16 (55.2%)	64 (50.8%)	0.671
Hypertension, *n* (%)	20 (69.0%)	89 (70.6%)	0.859
Diabetes, *n* (%)	14 (48.3%)	46 (36.5%)	0.241
Dyslipidemia, *n* (%)	11 (37.9%)	56 (44.4%)	0.523
Prior stroke, *n* (%)	9 (31.0%)	21 (16.7%)	0.077
LDL‑C (mmol/L), mean ± SD	0.99 ± 0.28	1.10 ± 0.96	0.469
HDL‑C (mmol/L), mean ± SD	2.40 ± 1.08	2.28 ± 0.81	0.576
Triglycerides (mmol/L), mean ± SD	1.70 ± 1.16	1.33 ± 0.66	0.106
Total cholesterol (mmol/L), mean ± SD	4.03 ± 1.33	3.69 ± 1.04	0.142
NIHSS score, median (IQR)	3 (1–4)	3 (1–5)	0.565
mRS score, median (IQR)	2 (1–3)	2 (1–3)	0.813
Intracranial stenosis 50–70%, *n* (%)	8 (27.6%)	33 (26.2%)	0.878
Intracranial stenosis ≥ 70%, *n* (%)	21 (72.4%)	93 (73.8%)	–
Extracranial carotid stenosis ≥ 50%, *n* (%)	9 (31.0%)	41 (32.5%)	0.876
Coronary stenosis ≥ 50%, *n* (%)	14 (48.3%)	69 (54.8%)	0.528

*BMI* body mass index, *LDL‑C* low-density lipoprotein cholesterol, *HDL‑C* high-density lipoprotein cholesterol, *NIHSS* National Institutes of Health Stroke Scale, *mRS* modified Rankin Scale

### Distribution of Calcification in Cardiovascular and Cerebrovascular Systems

Of the 155 patients, 136 (87.7%) had at least 1 calcific plaque in the cardiovascular and cerebrovascular systems. The intracranial artery was the most frequently affected segment for calcification, followed by the coronary artery, aortic arch, and extracranial carotid artery. The presence of intracranial calcium was significantly higher in patients with recurrent stroke than those without (96.6% vs. 69.0%, *p* = 0.002, Table 2), whereas it was not statistically significant at the site of the extracranial carotid artery, aortic arch, and coronary artery. In addition, the incidence of intracranial calcium increased with age (*p* < 0.001), and no significant difference was found in the presence of intracranial calcium between patients with moderate and severe intracranial stenosis (65.9% vs. 77.2%, *p* = 0.155), or between the ipsilateral side and contralateral side to the stroke (70.3% vs. 63.2%, *p* = 0.185, Supplementary Fig. 2).

**Table 2:** Distribution of calcification in cardiovascular and cerebrovascular systems

	Recurrence (*n* = 29)	No recurrence (*n* = 126)	*p* value
The presence of calcium			
Coronary artery, *n* (%)	22 (75.9%)	90 (71.4%)	0.631
Aortic arch, *n* (%)	23 (79.3%)	86 (68.8%)	0.262
Extracranial carotid artery, *n* (%)	23 (79.3%)	80 (63.5%)	0.104
Intracranial artery, *n* (%)	28 (96.6%)	87 (69.0%)	0.002

### Intracranial Calcium Patterns and Recurrent Ischemic Stroke

A total of 424 calcific plaques were found in intracranial arteries, of which 164 were SC and 260 were large calcific plaques. In the recurrence group, SC was found in 25 (86.2%) patients, including 8 patients with SC alone and 17 patients with both SC and large calcific plaques in intracranial arteries. In the non-recurrence group, SC was found in 51 (40.5%) patients, of whom 11 with SC alone and 40 with both SC and large calcific plaques (Table 3). Patients with intracranial SC were more likely to have recurrent ischemic stroke than those without (32.9% vs. 5.1%), with a hazard ratio of 7.03 (95% confidence interval, CI 2.44–20.23, *p* < 0.001). Furthermore, we compared the clinical characteristics according to the presence of intracranial SC. Patients with intracranial SC were older, more smokers, and had higher lipid levels (low-density lipoprotein cholesterol, triglycerides, and total cholesterol) than those without SC (Supplementary Table 1).

**Table 3:** Comparison of intracranial calcium patterns between patients with and without recurrent ischemic stroke

	Recurrence (*n* = 29)	No recurrence (*n* = 126)	HR (95% CI)	*p* value
Large calcific plaque only	3 (10.3%)	36 (28.6%)	2.98 (0.31–28.68)	0.344
SC only	8 (27.6%)	11 (8.7%)	17.34 (2.16–139.02)	0.007
Mixed	17 (58.6%)	40 (31.8%)	12.84 (1.71–96.57)	0.013

*SC* spotty calcium, *HR* hazard ratio, *CI* confidence interval

### Intracranial Calcium Density and Recurrent Ischemic Stroke

As shown in Table 4, the presence of very low-density calcium was higher in the recurrence group than in the non-recurrence group (72.4% vs. 37.3%), with the hazard ratio of 3.32 (95% CI 1.47–7.51, *p* = 0.001). On the per lesion level, large calcific plaque was denser than SC (1233.06 ± 297.39 HU vs. 687.93 ± 315.16 HU, *p* < 0.001, Supplementary Fig. 3).

**Table 4:** Comparison of intracranial calcium density between patients with and without recurrent ischemic stroke

	Recurrence (*n* = 29)	No recurrence (*n* = 126)	HR (95% CI)	*p* value
Very low-density calcium	21 (72.4%)	47 (37.3%)	3.32 (1.47–7.51)	0.001
Low-density calcium	14 (48.3%)	45 (35.7%)	1.43 (0.69–2.97)	0.209
High-density calcium	13 (44.8%)	54 (42.9%)	1.24 (0.59–2.60)	0.847
Very high-density calcium	16 (55.2%)	50 (39.7%)	2.02 (0.96–4.27)	0.128

*HR* hazard ratio, *CI* confidence interval

### Multivariable Cox Analysis

**Fig. 2:**
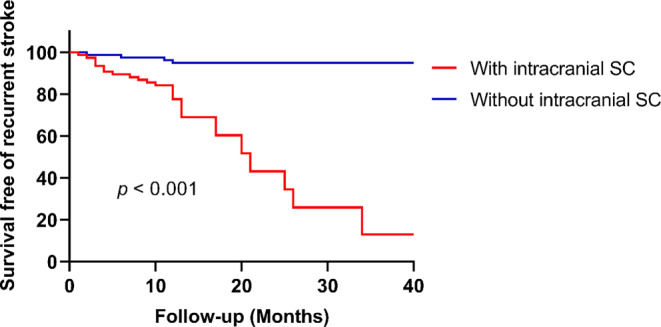
Kaplan-Meier curve for recurrent ischemic stroke stratified by the presence of intracranial SC. Patients without intracranial SC had a significantly higher recurrence-free rate than those with intracranial SC (log-rank *p* < 0.001). The X‑axis represents the time of follow-up in months. The Y‑axis represents the proportion of patients who survived free of recurrent ischemic stroke. *SC* spotty calcium

As shown in Fig. 2, the Kaplan-Meier analysis with the log-rank test showed that participants without intracranial SC had a significantly higher recurrence-free rate than those with intracranial SC (*p* < 0.001). The variables with *p* < 0.1 in the univariable analysis, including age, prior stroke, the presence of intracranial calcium, the presence of intracranial very low-density calcium, and the presence of intracranial SC, were adjusted in the multivariable Cox regression analysis. There was no multicollinearity between variables in the multivariable analysis (the tolerance value > 0.1 and the variance inflation factor was < 10 for each covariate, Supplementary Table 2). After adjusting for confounding factors, the presence of intracranial SC remained significantly associated with recurrent ischemic stroke (adjusted hazard ratio 5.35, 95% CI 1.32–21.69, *p* = 0.019; Table 5). The increased stroke recurrence risk associated with intracranial SC remained significant in patients with severe intracranial stenosis (Supplementary Table 3), while neither the presence of intracranial calcium nor the very low-density calcium were significantly associated with recurrent ischemic stroke (*p* > 0.05).

**Table 5:** Multivariable analyses of risk factors for recurrent ischemic stroke

	Multivariable analysis	
	HR (95% CI)	*p* value
Intracranial calcium	1.70 (0.16–18.04)	0.661
Very low-density calcium	0.89 (0.33–2.43)	0.820
Intracranial SC	5.35 (1.32–21.69)	0.019

*SC* spotty calcium, *HR* hazard ratio, *CI* confidence interval

## Discussion

The present prospective cohort study identified that the presence of intracranial SC and very low-density calcium were more prevalent in patients with recurrent ischemic stroke than in those without. After adjusting for confounding factors, intracranial SC remained an independent predictor of recurrent ischemic stroke. These findings suggest that intracranial SC should be included in the stroke risk stratification work-up in symptomatic ICAS patients.

It is noteworthy that patients with intracranial SC had an approximately five-fold increased risk of recurrent stroke compared with those without intracranial SC. This trend was also significant in patients with severe intracranial stenosis, which suggests that SC may play an important role in the pathological progression of ICAS. Although we cannot conclude that intracranial SC directly cause artery-to-artery embolism because of the uncertainty of the type of recurrent stroke, it is likely that intracranial SC leads to the recurrent stroke in ICAS patients through artery-to-artery embolism, regardless of hemodynamic instability. Recent cross-sectional studies showed that cervicocephalic SC was more frequently detected in patients with ischemic stroke compared to subjects with asymptomatic carotid atherosclerosis [19, 20]. Our findings provide further evidence that intracranial SC drives the recurrent risk of ischemic stroke in symptomatic ICAS patients, which will greatly contribute to advancing insights into the etiology and pathogenesis of ICAS.

We also noted that patients with intracranial SC were significantly older, were more often smokers, and had higher lipid profiles than those without intracranial SC. The progressive lipid depositions may be a result of the higher level of lipid profile and the co-occurrence of other cardiovascular risk factors. From a pathological point of view, SC often occurs around the plaque lipid pool and further stimulates the inflammatory response, thereby perpetuating the inflammatory cycle and leading to plaque instability [25, 26]. Besides, it has been shown that statins, the most widely used lipid-lowering treatment, could stabilize atherosclerotic plaques through increasing denser calcium and promoting macrocalcification, particularly in intensive statin treatment [27–29]. Consequently, more intensive lipid-lowering treatment should be justified in symptomatic ICAS patients with intracranial SC. Large clinical trials are warranted in the future to investigate the evolution of calcium patterns under the lipid-lowering treatment, which may have important clinical implications for guiding the secondary prevention of ICAS.

In the present study, very-low density calcium in the intracranial artery was not independently associated with the stroke recurrence. According to the Multi-Ethnic Study of Atherosclerosis (MESA), lower calcium density was associated with an increased risk of future major vascular events [14, 30]. However, this result was not verified in the Framingham study [31]. In our study, the density of each calcific plaque was directly measured and it was found that intracranial SC, rather than the very low-density calcium, may be more powerful for predicting recurrent ischemic stroke. We speculate that the effect of the very low-density calcium on future recurrent ischemic stroke may be offset by SC, eventually resulting in a null result.

Our study has several limitations. Firstly, this was a single-center study with a relatively small sample size and may have selection bias. These results need to be validated by a large sample multicenter study. Secondly, this study focused on the identification of calcium patterns and the measurement of calcium density, with limited information on other plaque compositional features. Advanced imaging techniques are needed to evaluate plaque compositions more precisely and quickly in the future. Finally, the vascular beds of posterior circulation were not evaluated in our study because some segments of vertebral arteries are shielded by the vertebrae. Also, patients who received endovascular treatment were excluded in our study. Both of these conditions may underestimate the real burden and prognosis of patients with ICAS.

## Conclusion

In patients with symptomatic ICAS, intracranial SC is an independent predictor of recurrent ischemic stroke. Our findings will further facilitate risk stratification and suggest that ICAS patients with intracranial SC may benefit from more aggressive treatment.

### Supplementary Information

Supplementary information showing additional methods and results: Supplementary Tables 1 to 3. Supplementary Figures 1 to 3.
