# Supplementary material for: Intracranial Spotty Calcium Predicts Recurrent Stroke in Patients with Symptomatic Intracranial Atherosclerotic Stenosis: A Prospective Cohort Study
Source: Clin Neuroradiol. 2023 Jun 7;33(4):985–92. doi: 10.1007/s00062-023-01299-7 (PMC10654160; doi:10.1007/s00062-023-01299-7)
Supplement: Supplementary file 1 — Supplementary information showing additional methods and results: Supplementary Tables 1 to 3. Supplementary Figures 1 to 3. [file 62_2023_1299_MOESM1_ESM.docx]

**Intracranial Spotty Calcium Predicts Recurrent Stroke in Patients with Symptomatic Intracranial Atherosclerotic Stenosis: A Prospective Cohort Study**

**Supplementary Material**

**Supplementary Table 1** Demographic and clinical characteristics of patients with and without intracranial SC

|  | With intracranial SC  (*n* = 76) | Without intracranial SC  (*n* = 79) | *p* value |
| --- | --- | --- | --- |
| Age (years), mean± SD | 61.45 ± 8.25 | 54.90 ± 13.45 | 0.012 |
| Male, *n* (%) | 56(73.7%) | 56(70.9%) | 0.697 |
| BMI (kg/m^2^), mean± SD | 25.18 ± 2.84 | 25.56 ± 3.45 | 0.463 |
| Smoking, *n* (%) | 46(60.5%) | 34(43.0%) | 0.029 |
| Hypertension, *n* (%) | 53(69.7%) | 56(70.9%) | 0.876 |
| Diabetes, *n* (%) | 33(43.4%) | 27(34.2%) | 0.238 |
| Dyslipidemia, *n* (%) | 35(46.1%) | 32(40.5%) | 0.486 |
| Prior stroke, *n* (%) | 17(22.4%) | 13(16.5%) | 0.352 |
| LDL-c (mmol/L), mean± SD | 2.50 ± 0.88 | 2.11 ± 0.82 | 0.005 |
| HDL-c (mmol/L), mean± SD | 1.03 ± 0.34 | 1.12 ± 1.19 | 0.827 |
| Triglycerides (mmol/L), mean± SD | 1.64 ± 0.99 | 1.17 ± 0.40 | 0.001 |
| Total cholesterol (mmol/L), mean± SD | 3.95 ± 1.18 | 3.56 ± 1.00 | 0.031 |
| NIHSS score, median (IQR) | 3(0-5) | 2(1-5) | 0.669 |
| mRS score, median (IQR) | 2(1-3) | 2(1-3) | 0.245 |

*SC* spotty calcium, *BMI* body mass index, *LDL-c* low density lipoprotein-cholesterol, *HDL-c* high density lipoprotein-cholesterol, *NIHSS* National Institutes of Health Stroke Scale, *mRS* modified Rankin Scale

**Supplementary Table 2** The analysis of multicollinearity between variables with *p* < 0.1 in the univariate analysis

|  | Tolerance | Variance inflation factor |
| --- | --- | --- |
| Age | 0.631 | 1.584 |
| Prior stroke | 0.956 | 1.046 |
| The presence of intracranial calcium | 0.415 | 2.411 |
| The presence of very low-density calcium | 0.455 | 2.196 |
| The presence of intracranial SC | 0.456 | 2.194 |

*SC* spotty calcium

**Supplementary Table 3** Association of intracranial SC with recurrent ischemic stroke in patients with severe ICAS

|  | Univariable analysis | | Multivariable analysis | |
| --- | --- | --- | --- | --- |
|  | HR (95% CI) | *p* value | HR (95% CI) | *p* value |
| Intracranial SC | 7.51(1.73-32.51) | 0.007 | 6.47(1.17-35.93) | 0.033 |

*ICAS* intracranial atherosclerotic stenosis, *SC* spotty calcium, *HR* hazard ratio, *CI* confidence interval

**
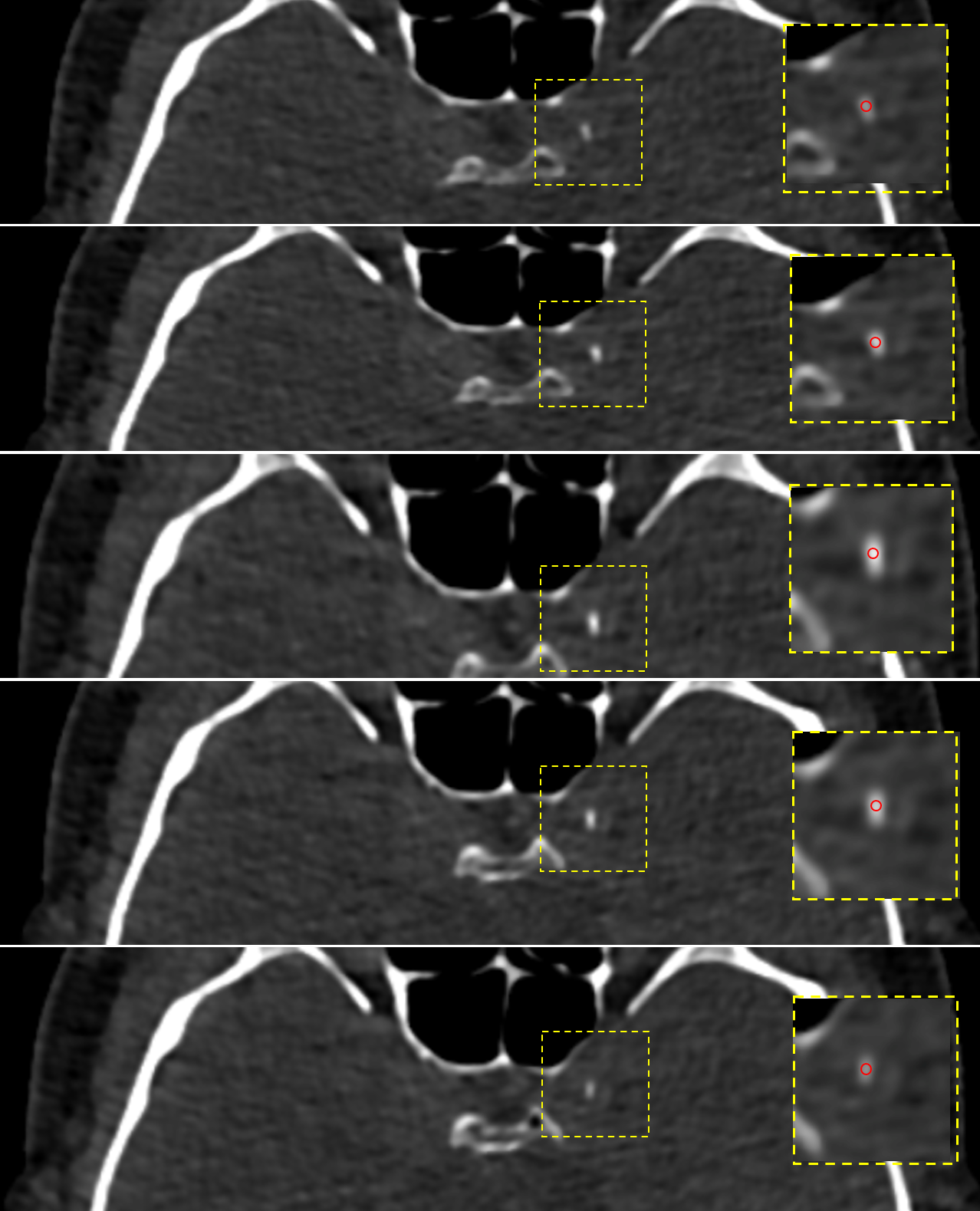
**

**Supplementary Fig. 1** Example of calcium density measurement. The calcium density was measured by manually placing the ROI on all planes of the entire calcified plaque and defined as the highest CT value for each calcified plaque


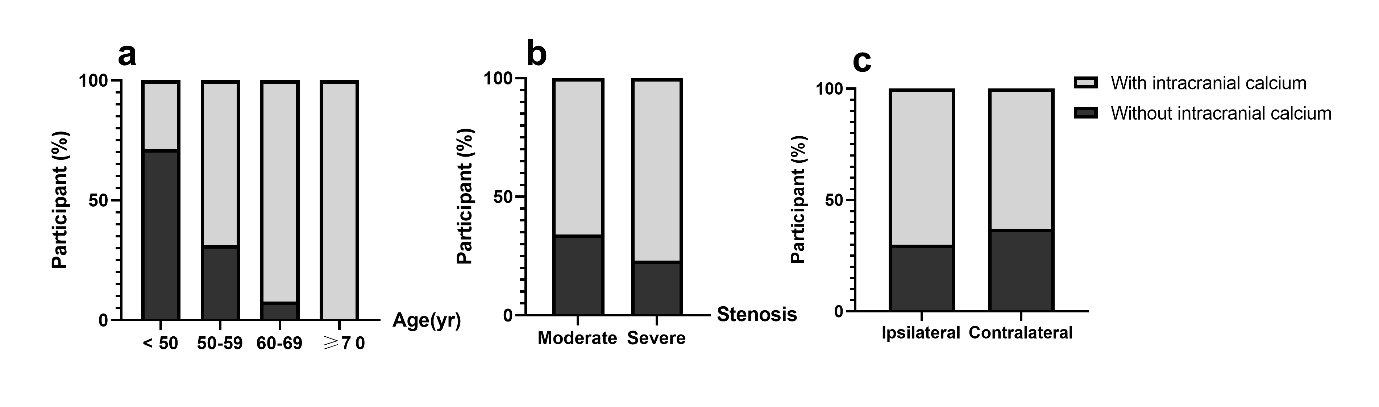


**Supplementary Fig. 2** Intracranial calcium characteristics. The presence of intracranial calcium stratified by age (a) and the severity of intracranial stenosis (b), as well as in ipsilateral and contralateral side to the stroke (c)

**
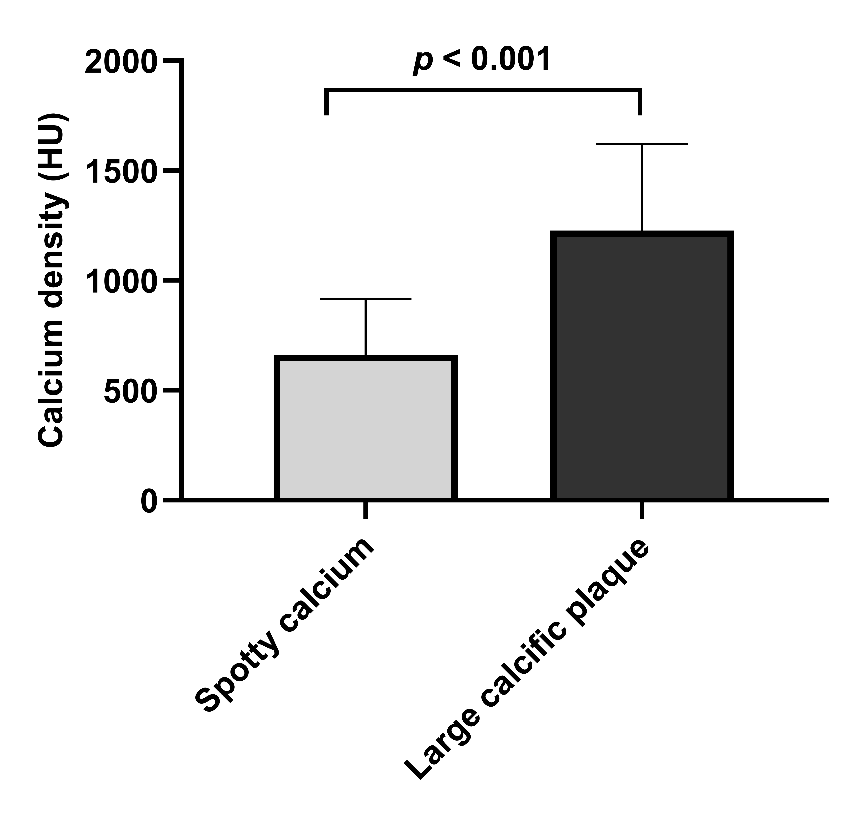
**

**Supplementary Fig. 3** Comparison of calcium density between spotty calcium and large calcific plaque
